# Supplementary material for: Evaluation of the Growth-Inhibitory Spectrum of Three Types of Cyanoacrylate Nanoparticles on Gram-Positive and Gram-Negative Bacteria
Source: Membranes (Basel). 2022 Aug 15;12(8):782. doi: 10.3390/membranes12080782 (PMC9414559; doi:10.3390/membranes12080782)
Supplement: Supplementary file 1 [file membranes-12-00782-s001.zip › membranes-1835073-supplementary.pdf]

Supplementary Material

1 Supplementary Figures

1.1 Supplementary Figures

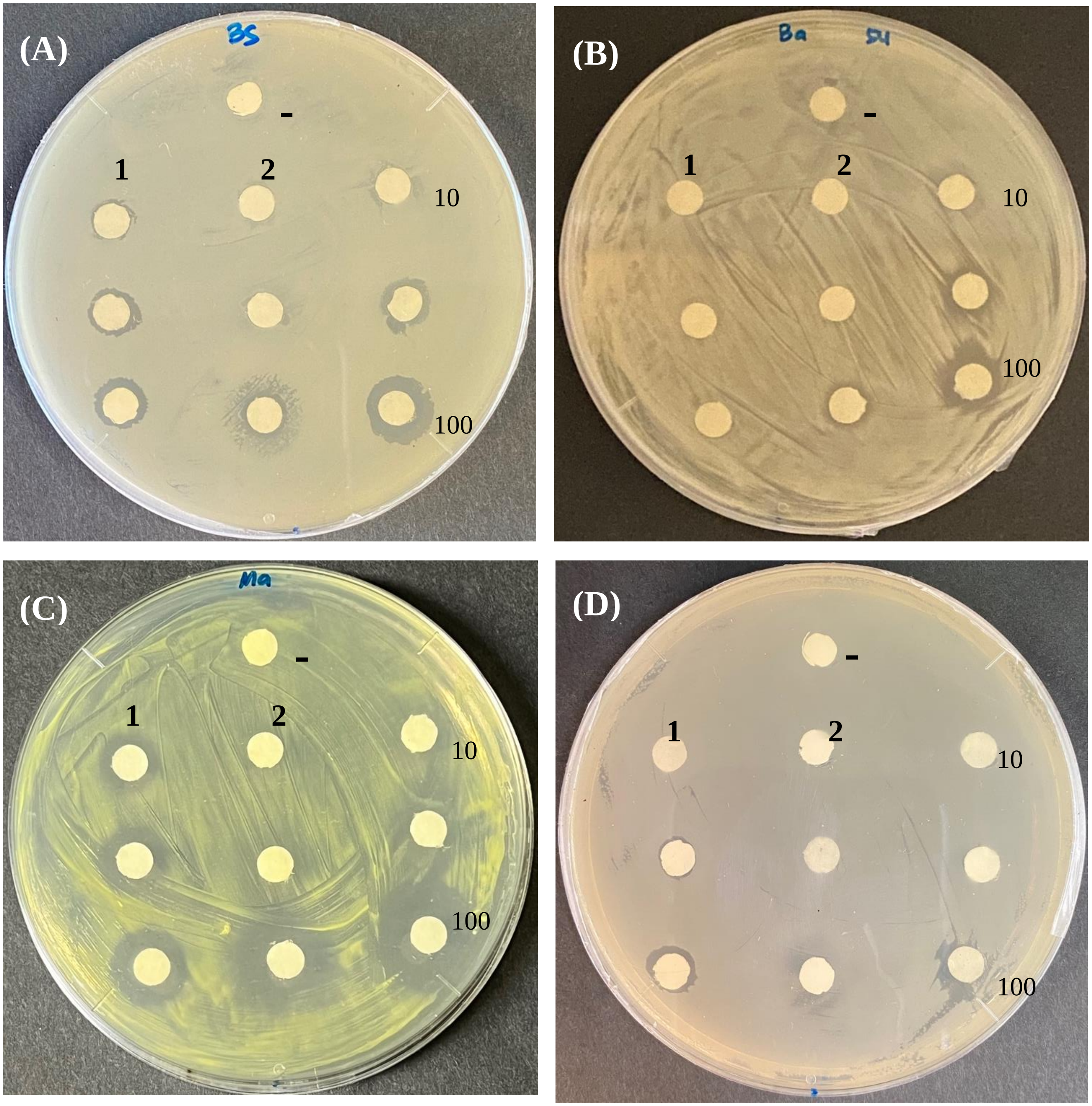

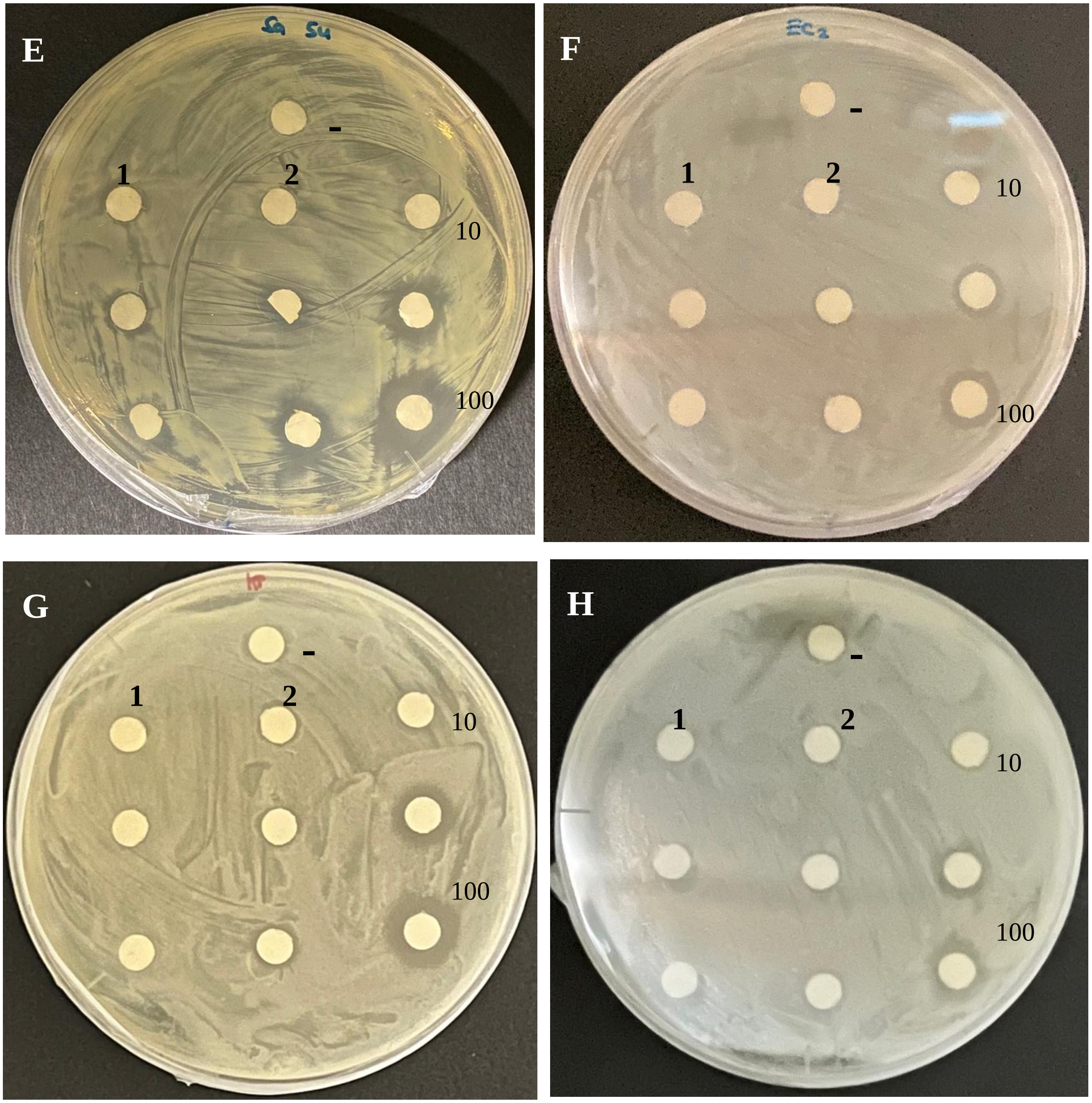
E
-
1
2
10
100
F
EC2
-
1
2
10
100
G
-
1
2
10
100
H
-
1
2
10
100

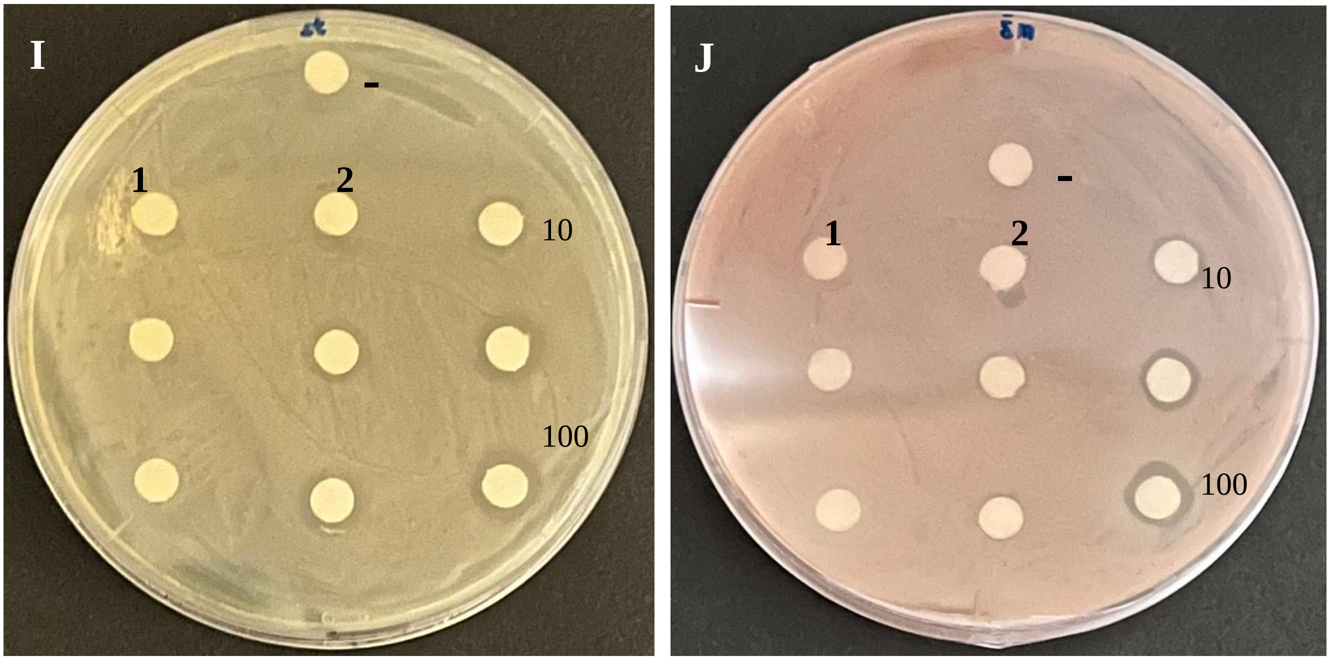

Supplementary Figure S1. Non cell growth zone appeared around paper disks containing iBCA-NPs, EECA-NPs, or ECA-NPs. Gram-positive bacteria: A, *Bacillus subtilis*; B, *Brevibacillus agri*; C, *Microbacterium aurum*; D, *Propobacterium acnes*; E, *Staphylococcus aureus*; Gram-negative bacteria: F, *Escherichia coli*; G, *Klebsiella pneumoniae*; H, *Pseudomonas aeruginosa*; I, *Salmonella typhimurium;* J, *Serratia marcescens*.

(-), control paper disk contains Tween80; 1, paper disk contains iBCA-NPs; 2, paper dis contains EECA-NPs; 3, paper disk contains ECA-NPs.

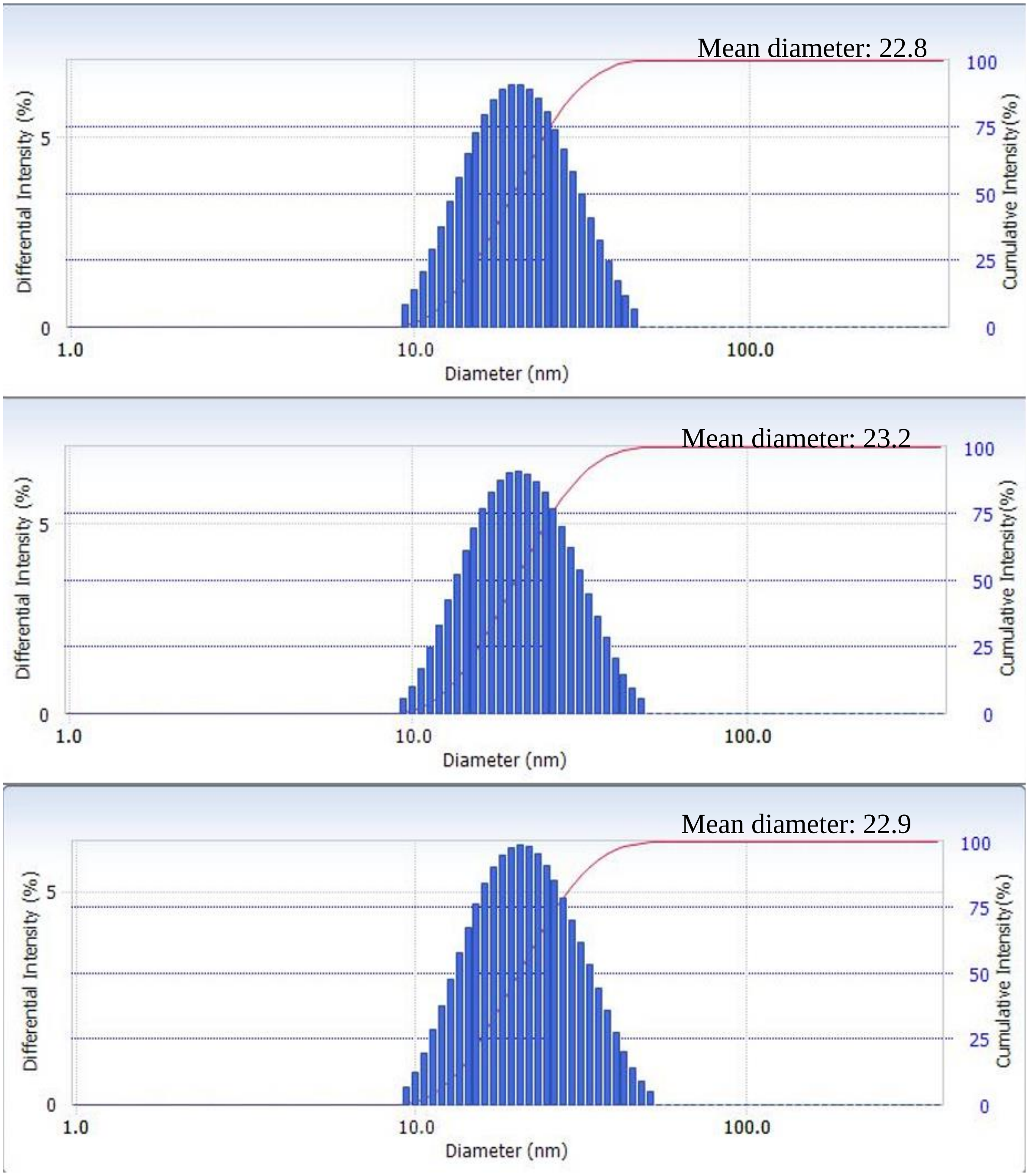

Supplementary Figure S2. Particle size distribution histogram of iBCA-NPs used in the present study.

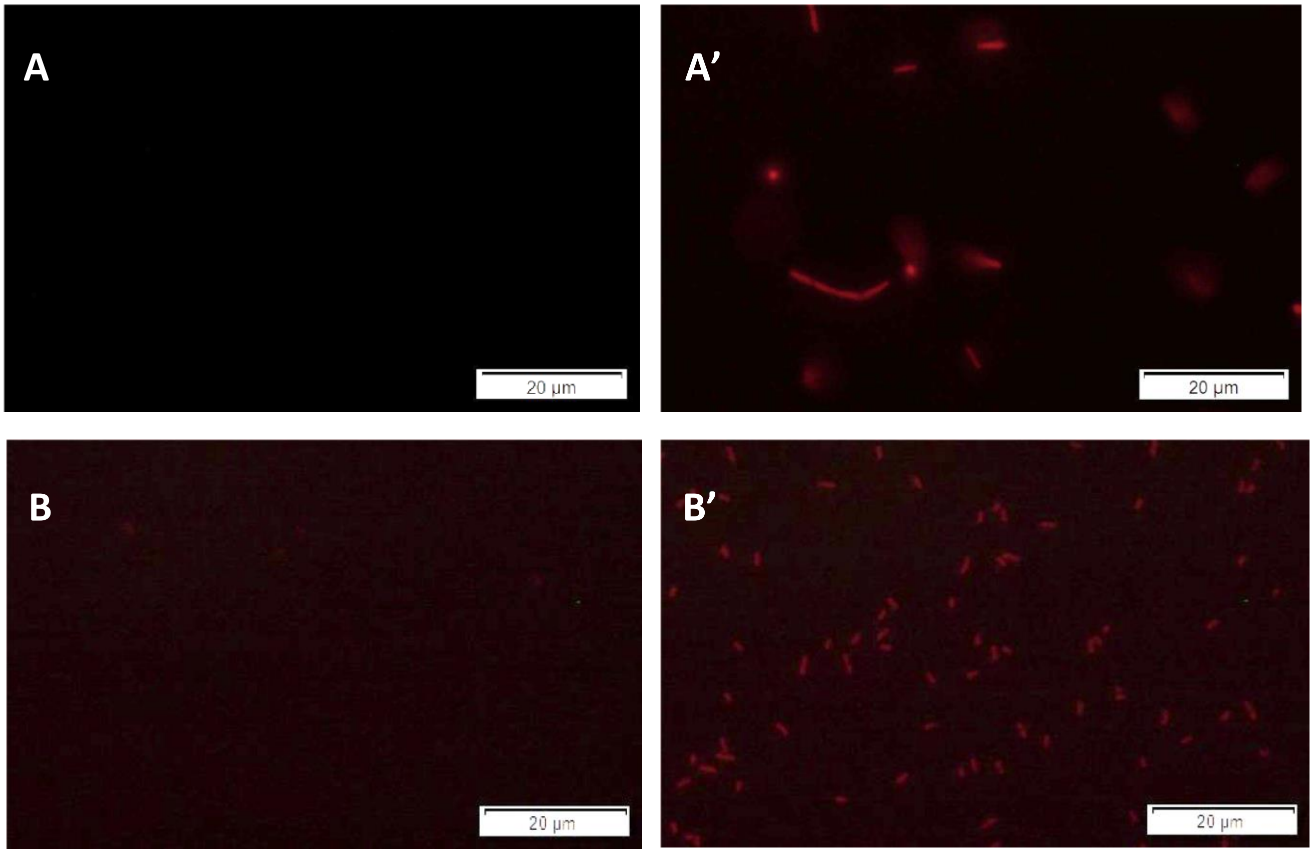

Supplementary Figure S3. Fluorescence microscopy of cells after 2 h exposure with 100 mg/L of ECA-NPs and untreated cells. Fluorescence probe PI was used to detect the cell membrane damage. (**A**), *Bacillus subtilis* (Gram-positive); (**B**), *Escherichia coli* (Gram-negative); alphabet, fluorescence of untreated cells; alphabet with quotation mark (**'**), fluorescence image of treated samples.
